# INTEREST GROUP SESSION—RURAL AGING: INNOVATIONS THAT SUPPORT RURAL OLDER ADULTS’ HEALTH AND WELL-BEING: MODELS, NETWORKS, CASE STUDIES, REFLECTIONS

**DOI:** 10.1093/geroni/igz038.2035

**Published:** 2019-11-08

**Authors:** Roger O’Sullivan, Lyn Holley, Marc A Guest

**Affiliations:** 1 Institute of Public Health in Ireland, Belfast & Dublin, Ireland; 2 University of Nebraska, Omaha, United States; 3 University of Kentucky, Lexington, Kentucky, United States

## Abstract

Access of rural older people to health and wellness services is limited and becoming progressively more limited as trends toward increasing centralization of Government and private services continue. “Top-down” or urban centric models for rural service delivery often miss context essential to effectiveness and sustainability. In this symposium, each presenter in this multidisciplinary group of researchers presents innovative, community-based interventions that address these challenges using different methodologies and in respect to different needs Maiden (Psychology) compares the utilization of mental health services by rural older adults over time with their need for such services. Through the lens of social gerontology Holley examines networks of support that have intersected successfully to generate local solutions to unmet needs of rural-dwelling older adults. Crowther and Ford within a nursing and care context explore community-based models that draw upon the role of culture to integrate care for rural older adults. Katz, from an adult development perspective, reports on an educational game-intervention developed to reduce cognitive decline which is tailored specifically for older adults in rural areas. Wiese presents evidence from a pilot home-based approach that demonstrates a model for increasing rates of AD detection and treatment in a rural retired farm worker community in Florida. Our discussant, an emerging scholar in the field of rural gerontology, will reflect on the major themes that emerge from these multidisciplinary perspectives, especially the role of intersecting networks in community-based innovations and rural aging.

